# Investigation of Association between PFO Complicated by Cryptogenic Stroke and a Common Variant of the Cardiac Transcription Factor GATA4

**DOI:** 10.1371/journal.pone.0020711

**Published:** 2011-06-06

**Authors:** Mahdi Moradi Marjaneh, Edwin P. Kirk, Maximilian G. Posch, Cemil Ozcelik, Felix Berger, Roland Hetzer, Robyn Otway, Tanya L. Butler, Gillian M. Blue, Lyn R. Griffiths, Diane Fatkin, Jeremy J. Martinson, David S. Winlaw, Michael P. Feneley, Richard P. Harvey

**Affiliations:** 1 The Victor Chang Cardiac Research Institute, Darlinghurst, Australia; 2 Faculty of Medicine, University of New South Wales, Kensington, Australia; 3 Department of Medical Genetics, Sydney Children's Hospital, Randwick, Australia; 4 The Experimental and Clinical Research Center (ECRC), Charité – Universitätsmedizin, Berlin, Germany; 5 Deutsches Herzzentrum Berlin, Berlin, Germany; 6 Heart Centre for Children, The Children's Hospital at Westmead, Westmead, Australia; 7 Sydney Medical School, University of Sydney, Sydney, Australia; 8 Genomics Research Centre, Griffith University, Gold Coast, Australia; 9 Cardiology Department, St Vincent's Hospital, Darlinghurst, Australia; 10 University of Pittsburgh, Pittsburgh, Pennsylvania, United States of America; VIB & Katholieke Universiteit Leuven, Belgium

## Abstract

Patent foramen ovale (PFO) is associated with clinical conditions including cryptogenic stroke, migraine and varicose veins. Data from studies in humans and mouse suggest that PFO and the secundum form of atrial septal defect (ASDII) exist in an anatomical continuum of septal dysmorphogenesis with a common genetic basis. Mutations in multiple members of the evolutionarily conserved cardiac transcription factor network, including GATA4, cause or predispose to ASDII and PFO. Here, we assessed whether the most prevalent variant of the GATA4 gene, S377G, was significantly associated with PFO or ASD. Our analysis of world indigenous populations showed that GATA4 S377G was largely Caucasian-specific, and so subjects were restricted to those of Caucasian descent. To select for patients with larger PFO, we limited our analysis to those with cryptogenic stroke in which PFO was a subsequent finding. In an initial study of Australian subjects, we observed a weak association between GATA4 S377G and PFO/Stroke relative to Caucasian controls in whom ASD and PFO had been excluded (OR = 2.16; p = 0.02). However, in a follow up study of German Caucasians no association was found with either PFO or ASD. Analysis of combined Australian and German data confirmed the lack of a significant association. Thus, the common GATA4 variant S377G is likely to be relatively benign in terms of its participation in CHD and PFO/Stroke.

## Introduction

Stroke is the third leading cause of death [1] and the major cause of serious, long-standing disability in the United States [2] affecting about 800,000 individuals each year [3]. Among cases with ischemic stroke, 18–42% have no identifiable cause and are termed cryptogenic [4]–[7]. A well-recognised risk factor for cryptogenic stroke, particularly in young patients, is patent foramen ovale (PFO) [8], a common cardiac atrial septal variant in which the septum primum fails to fuse with the septum secundum after birth, leading to the potential for inter-atrial communication. Approximately 50% of stroke patients younger than 55 years have PFO [9]–[12], which is at least double the prevalence in the general population [13], [14]. A likely mechanism for involvement of PFO in stroke is “paradoxical embolism”, in which a venous thrombus crosses the atrial septum and enters the systemic circulation, although an abnormal septum may also be thrombogenic [8]. The severity of PFO in the normal population is variable, ranging from small pinhole openings to large tunnel-shaped corridors, and only ∼1% of PFO show an inter-atrial communication of ≥10 mm [14]. However, PFO in patients with stroke are generally large (>2 mm) [15], [16] and often associated with increased mobility or aneurysm of the fossa ovalis membrane, the remnant of embryonic septum primum [17], [18].

PFO is also associated with other conditions. A number of studies show a higher prevalence of PFO in migraineurs with aura [19], [20], [21] and a dramatic lessening of migraine attacks after PFO closure [22], [23], suggesting that PFO acts as a risk factor for migraine. However, not all studies found such an association [24]. Neurological decompression sickness is also more common in divers with PFO (and ASD) [25] and a recent study found that the prevalence of PFO was higher in patients with symptomatic varicose veins due to great saphenous incompetence [26].

There is no conclusive data on the underlying causes of PFO, although there is good evidence that genetic factors are involved [27]–[31]. The newly reported association between PFO and varicose veins [26], a common and highly hereditary vascular abnormality [32], also indicates genetic contribution to PFO.

ASD is a more severe atrial septal abnormality than PFO [33]. In the secundum form of ASD (ASDII), there is incomplete coverage of the fossa ovalis by the atrial septum primum, leading to persistent shunting of blood from left to right atrium. ASDII accounts for approximately 7% of all cases of congenital heart defect (CHD) [34], [35].

A link between ASD and PFO has not been formally established in humans, although the largest and most complex PFO can be difficult to distinguish from ASD and are often regarded as *form fruste* ASD. This relationship is also strongly suggested by family studies, including those bearing mutations in cardiac transcription factor genes. Mutations in TBX20, for example, are associated with defects in septation including ASD and PFO with permanent left-to-right shunt [27]. Heterozygous TBX20 mutant mice show an increased background prevalence of PFO and septal dysmorphogenesis, as well as a genetic predisposition to ASD [30]. Mutations in the homeodomain factor NKX2-5, a key transcriptional regulator of cardiac development, cause familial ASD in humans, and in mice lead to a high prevalence of PFO correlating with the severe end of a spectrum of atrial septal dysmorphogenesis [31], [36]–[42]. The prevalence of PFO in mice is also highly genetic background-dependent. Collectively, these data suggest that ASD and PFO exist in an anatomical continuum with a common genetic basis. Furthermore, the genetic underpinnings of PFO are likely to be highly heterogeneous. Indeed, analysis of quantitative trait loci (QTL) contributing to atrial septal defects or PFO in inbred strains of mice identified 13 significant or suggestive QTL [43].

GATA4 belongs to the conserved GATA family of zinc finger transcription factors which play an important role in mammalian cardiac lineage specification and morphogenesis [44]. Mutations in GATA4 are associated with CHD in humans, particularly ASDII [45]. Thus, it is apparent that perturbations in multiple members of the evolutionarily conserved cardiac transcription factor network cause or predispose to ASDII and PFO. The most prevalent variant of the GATA4 gene is an A>G non-synonymous transition at position c.1647 resulting in a serine to glycine change at amino acid 377 (GATA4 S377G) with a minor allele frequency of 0.11 [46]. Although this is a conservative amino acid change, the polymorphism is located within the C-terminal GATA4 domain which is highly conserved in vertebrate species and essential for GATA4 transcriptional activity [45], [47], [48]. In vitro functional analysis using the alpha myosin heavy chain promoter revealed that GATA4 S377G has a significantly enhanced transcriptional activity compared to wildtype GATA4 [49]. Although S377G showed no significant association with migraine [50], an association with CHD including ASD and PFO has not been characterized so far.

Here, we address whether there is a significant association between this GATA4 variant and ASD or PFO in patients unselected for family history. To focus on the more severe end of the PFO spectrum, PFO subjects comprised largely patients with cryptogenic stroke in whom PFO was subsequently found by echocardiography.

## Materials and Methods

### Global distribution of S377G

The distribution of S377G allele (dbSNP ref. 3729856) was determined in different indigenous populations [51] (Table 1) using Fluorescence Polarisation [52].

**Table 1:** S377G allele distribution in indigenous human populations.

		Totaln	Wild type^1^n (%)	S377G Heterozygosity^1^n (%)	S377G Homozygosity^1^n (%)	S377G Allele Frequency^1^%
**Caucasian**	Caucasian US	480	382 (79.6)	88 (18.3)	10 (2.1)	11.3
	UK	42	27 (64.3)	13 (31)	2 (4.8)	20.2
	Australia^2^	391	301 (77)	83 (21.2)	7 (1.8)	12.4
	Cyprus	37	27 (73)	9 (24.3)	1 (2.7)	14.9
	Russian Caucasus	112	84 (75)	26 (23.2)	2 (1.8)	13.4
	Russia	47	33 (70.2)	14 (29.8)	0 (0)	14.9
**Asian**	Yemen	89	64 (71.9)	24 (27)	1 (1.1)	14.6
	India/Pakistan	111	94 (84.7)	17 (15.3)	0 (0)	7.7
	Hong Kong	57	57 (100)	0 (0)	0 (0)	0
	Taiwan	92	92 (100)	0 (0)	0 (0)	0
**African**	Madagascar	117	116 (99.1)	1 (0.9)	0 (0)	0.4
	Central African Republic	44	44 (100)	0 (0)	0 (0)	0
**Pacific Islander**	Papua New Guinea	88	88 (100)	0 (0)	0 (0)	0

1 Reflects the prevalence of the A>G single nucleotide polymorphism that causes the S377G amino acid change.

2 The same population were used as “population controls” in the Australian cohort.

### Ethics Statement

All human experiments in the Australian study were carried out under Human Research Ethics Committee approval from the South East Health Research Ethics Committee – Eastern Division, the St Vincent's Hospital Research Ethics Committee and the Children's Hospital at Westmead Research Ethics Committee.

The German study protocol was approved by the Institutional Review Board of the Charité-Universitätsmedizin Berlin, Germany.

Written informed consent was obtained from all participants (or parental/legal guardian on behalf of the children participating in the study).

### Australian Study

Subjects were recruited from Sydney hospitals from 1982 to 2004 and those of Caucasian descent were selected for the study. Adult ASD and CHD patients were recruited mostly from St. Vincent's Hospital and St. Vincent's Private Hospital (SVHs), while children were from the Sydney Children's Hospital (SCH) and the Children's Hospital at Westmead (CHW). Most were recruited prospectively during outpatient services and were unselected for family history or particular cardiac anomalies. PFO and Stroke patients were recruited prospectively from St Vincent's Hospital and St Vincent's Private Hospital after referral to echocardiography services for a variety of indications including stroke or transient ischemic episode (TIA). Allocation to case (PFO with cryptogenic stroke; n = 58) and control (PFO without stroke/TIA, n = 29; stroke/TIA without PFO/ASD, n = 66) groups occurred retrospectively. Cases consisted of 58 individuals under investigation for cryptogenic stroke in whom PFO was demonstrated by transesophageal echocardiography (TEE). In control groups, PFO was either absent or an incidental finding, as also determined by TEE.

Clinical evaluation included medical history, 12-lead electrocardiography and transthoracic echocardiography and/or TEE with intravenous saline contrast injection during the strain and release phases of the Valsalva maneuvre. Two additional control groups were also assessed. TEE controls recruited from St. Vincent's Hospital (n = 113) had undergone TEE for a range of indications and were reported to have structurally normal hearts, in particular an intact atrial septum. The second group (population controls; n = 391) were Australian Caucasians in generally good health (no stroke or known heart disease) although unselected for septal status [50]. The PFO/Stroke group was restricted to patients with cryptogenic stroke. Since atrial fibrillation (AF) is a known cause of stroke, all stroke patients had Holter monitor evaluation unless there was prior evidence in their medical history for AF. We excluded all patients with AF from the PFO/Stroke group. Stroke/no PFO/ASD were also evaluated by Holter monitor as part of their clinical evaluation, although those with AF were included in the study.

Initially, GATA4 coding exons were amplified by polymerase chain reaction (PCR) from 100 ng of leukocyte DNA, purified with PCR Cleanup Plates (Millipore) and sequenced using Big Dye Terminator v3.1 kit (Applied Biosystems) and ABI PRISM® 3700 DNA Analyser. Subsequently, GATA4 S377 status was confirmed in all samples, including population and TEE controls, using a commercial SNP analysis platform (Genera Biosystems). Chi-square or the Fisher's exact test was used to detect differences between allele frequencies.

### German study

A vast majority of individuals were of Caucasian origin and recruited from Berlin hospitals between 2005 and 2007. There were three Arab patients who were included in the study since the allele frequency of S377G is similar in both Arab and Caucasian populations (Table 1). Ninety-six and 95 patients had isolated ASDII and isolated PFO, respectively. ASDII probands represent a subgroup of a previously published CHD cohort included in genetic screening [53]. Most PFO probands (91%) had been admitted for interventional PFO closure after experiencing one or more thromboembolic events. All 191 cases were recruited at the Department of Pediatric Cardiology, Deutsches Herzzentrum Berlin, and Adult Cardiology Department at the Charité - Universitätsmedizin Berlin, Campus Virchow (CVK).

All patients were characterized by physical examination, 12-lead electrocardiography and TEE. Ninety-six patients attending the Department of Cardiology CVK were used as controls after exclusion of ASD and PFO by contrast-enhanced TEE after Valsalva maneuvre. All ASDII and PFO patients underwent 24 hour Holter monitor. AF was found in none of the PFO patients and all were designated as having cryptogenic stroke.

PCR fragments including the S377G variation were analysed by single stranded conformational polymorphism (SSCP). Differential migration patterns allowed a secure distinction of all three possible genotypes (AA, AG, GG) on 12% Polyacrylamide gels at 20°C. All three genotypes were confirmed in 12 samples by direct DNA sequencing. Discrete variables were expressed as counts or percentages and compared using chi-square test without Yates correction. Fisher's exact test was employed for association of counts of which one subgroup was smaller than four. Continuous variables were checked for Gaussian distribution and expressed as mean value ± standard deviation and were compared by use of unpaired, two sided t-test.

## Results

### Global distribution of GATA4 S377G

The global distribution of GATA4 S377G was determined by analysis of DNA from world indigenous populations [51]. European, Australian, American and Middle Eastern Caucasians had the highest allele frequency (11.3–20.2%), while Indians/Pakistanis had a lower frequency (7.7%) (Table 1). By contrast, Africans, East Asians and Pacific Islanders showed a very low frequency (0–0.4%). Allele frequency for all populations was in Hardy-Weinberg equilibrium. These data suggest a relatively recent and Caucasian origin for S377G, potentially in a single individual among Neolithic farming populations from the Near East before their expansion into Europe within the last 10,000 years [54]. Further comparisons of S377G frequency were restricted to Caucasian subjects.

### Australian study

In Table 2, baseline demographics and relevant clinical data are tabulated. As ASD and PFO likely exist in an anatomical continuum with a common genetic basis, we also included ASD subjects (n = 129) which were a mixture of children and adults (mean age 21.6 yrs) and the majority had pure ASDII. Other CHD subjects (n = 109) which served as controls, were mostly children (mean age 3.9 yrs) and the majority had isolated ventricular septal defect (VSD), VSD with minor abnormalities, VSD with outflow tract defects including tetralogy of Fallot, pulmonary atresia, transpositions or double outlet right ventricle (DORV). Both ASD and Other CHD subjects were unselected for family history, although 26.6% and 11%, respectively, had a known family history of CHD.

**Table 2:** Clinical characteristics of Australian cohort.

	ASD^1^(n = 129)	CHD controls^2^(n = 109)	PFO		Stroke/no PFO/ASD^5^(n = 66)	TEE Controls (no PFO/ASD/Stroke)^6^(n = 113)	Caucasian Population Controls(n = 391)
			With Stroke^3^(n = 58)	Without Stroke^4^(n = 29)			
**Age at Study** 7 **(yrs)**	0–77(mean:21.6)	0.1–44.3(mean:3.9)	19–86(mean:51.3)	38–88(mean:65.9)	29–87(mean:65.9)	21–89(mean:63.3)	16–84(mean:53.1)
**Male (%)**	54 (41.9)	69 (63.3)	32 (55.2)	20 (69.0)	44 (66.7)	66 (58.4)	200 (50)
**Family History of CHD** **n/total (%)** 7	34/128 (26.6)	12/109 (11.0)	6/56 (10.7)	4/27 (14.8)	2/65 (3.1)	12/113 (10.6)	N/A
**Severe Atheroma** **n/total (%)** 8	N/A	N/A	0/43 (0.0)	3/29 (10.3)	18/66 (27.3)	18/109 (16.5)	N/A
**Atrial Fibrillation** **n/total (%)**	N/A	N/A	0/58 (0.0)	N/A	13/66 (19.7)	N/A	N/A

1 Includes 2 APVD, 2 LSVC, 2 Coarctation of the aorta, 2 Pulmonary Stenosis, 1 LSVC & Pulmonary Stenosis, 1 PDA, 3 MVP.

2 Includes 26 isolated VSD, 17 VSD with minor abnormalities, 1 functional single ventricle and 64 VSD with malformations of outflow tracts - these include 34 with Tetralogy/pulmonary atresia, 15 with transposition/DORV and 15 with other malformations.

3 Includes 1 Ebstein's Anomaly, 2 MVP, 1 prosthetic pulmonary valve, 1 prosthetic mitral valve.

4 Includes 1 Quadri-leaflet Aortic Valve, 3 MVP, 3 prosthetic AV, 1 prosthetic AV&MVR.

5 Includes 1 BAV, 4 MVP, 4 prosthetic AV, 2 MVR.

6 Includes 1 Ebstein's Anomaly, 8 BAV, 1 BAV & Coarctation of the Aorta, 1 BAV & MVR, 1 Sick Sinus Syndrome, 1 PDA, 1 aortic root replacement, 9 MVP, 3 prosthetic MV, 6 prosthetic AV, 2 prosthetic AV&MV, 1 MVR, 1 prosthetic AV & MVR, 1 MVR & tricuspid VR.

7 Family history was unknown for a small number of patients.

8 Grade of atherosclerosis was unknown for 14 cases with PFO and stroke, 2 cases with PFO and without stroke and 4 TEE controls.

Within PFO/stroke cases (n = 58), the mean age was 51.3 yrs, some 12–14 yr younger than in our three adult control groups in which septal status was known (PFO without Stroke, Stroke without PFO/ASD, and TEE Controls), suggesting over-representation of patients with premature stroke. No patient had severe atheroma or AF. Only a minority (10.7%) had a family history of CHD. No significant gender bias was evident in either cases or controls.

In all study populations, GATA4 S377G allele distribution was in Hardy-Weinberg equilibrium. Prevalence of heterozygosity and allele frequency varied somewhat, although not significantly, between case and control groups (Table 3). In healthy Population Controls, which were unselected for atrial septal status (n = 391), the allele frequency was 12.4%, in good agreement with other world Caucasian populations (Table 1). The frequency was not significantly different in other CHD subjects, those with PFO without Stroke or Stroke without PFO/ASD. However, allele frequency in our gold standard TEE Controls who did not have stroke and in whom ASD and PFO had been specifically excluded (n = 113), was lower at 9.3%. Although this fell short of significance when compared to Population Controls (p = 0.20), it would be the expected trend if S377G played a causative role in PFO at the severe end of the spectrum.

**Table 3:** GATA4 genotypes of Australian cohort.

		ASD(n = 129)	CHD controls(n = 109)	PFO		Stroke/no PFO/ASD(n = 66)	TEE Controls (no PFO/ASD/Stroke)(n = 113)	Population Controls(n = 391)
				With Stroke(n = 58)	Without Stroke(n = 29)			
**GATA4 S377G**	**Heterozygous (%)**	18 (14)	24 (22.0)	15 (25.9)	8 (27.6)	16 (24.2)	17 (15.0)	83 (21.2)
	**Homozygous (%)**	3 (2.3)	3 (2.8)	3 (5.2)	0 (0.0)	0 (0.0)	2 (1.8)	7 (1.8)
	**Allele Frequency**	9.3	13.8	18.1^1^	13.8	12.1	9.3	12.4

1 p = 0.02 compared to TEE Controls; Odds ratio 2.16.

Allele frequency was also low in ASD subjects (9.3%), indicating no role for S377G in causation of ASDII. The differences between the ASD group and controls were not statistically significant. However, an excess of S377G was observed in cases with PFO and stroke, with an allele frequency of 18.1% (p = 0.02 comparing to the TEE control group).

### German study

We also assessed S377G allele frequency independently in German Caucasians with ASD and PFO compared to the TEE controls. Basic demographics and clinical information from ASD and PFO subjects are shown in Table 4. The ASD group (n = 96) comprised children and adults (aged 2.9 yrs to 75.9 yrs) who were mostly female (70.5%). This female preponderance of ASD is consistent with a previous epidemiologic report [55]. PFO probands (n = 95) were all adults (mean age 52.9) who mostly had been admitted subsequent to neurologic events (91.2%). During TEE, atrial septal aneurysm (ASA) was detected in a relatively high number of ASDs and PFOs (37.4% and 40.7% respectively). It is known that ASA is often associated with other atrial septal defects, in particular ASDII and PFO, and is also a risk factor for stroke [56], [57], [58]. More than two thirds of ASD subjects had an ECG pattern of right bundle branch block (RBBB) and about 14% had atrioventricular block (AVB). In the PFO group, RBB was found in just 20% and one patient had AVB.

**Table 4:** Clinical characteristics^1^ of German cohort.

	ASDII^2^(n = 96)	PFO/Stroke^3^(n = 95)
**Age at study** **(yrs)**	2.9–75.9(mean:38.6)	26.3–74.4(mean:52.9)
**Male (%)**	28 (29.5)	50 (54.9)
**Family history of CHD** **n/total (%)**	8/42 (19.2)	1/58 (1.7)
**Neurologic event (%)** 4	9 (9.9)	83 (91.2)
**Migraine (%)**	3 (3.3)	6 (6.6)
**Defect closure (%)** 5	89 (98.9)	91 (100)
**Atrial septal aneurysm (%)**	34 (37.4)	37 (40.7)
**AVB (%)** 6	13 (14.3)	1 (1.1)
**iRSB/RSB (%)** 7	68 (73.9)/6 (6.5)	19 (20.0)/2 (2.1)
**Atrial Fibrillation (%)**	3 (3.3)	0 (0)

1 Family history was unknown for 54 cases with ASDII and 37 cases with PFO. Other Clinical characteristics were unknown for a small number of patients.

2 All ASDII cases were free of any other cardiac malformation.

3 All PFO cases were free of any other cardiac malformation. All were designated as having cryptogenic stroke.

4 Neurologic event includes thromboembolic stroke, PRIND (prolonged reversible ischemic neurologic deficit) and TIA.

5 Defect closure includes interventional closure and surgical closure.

6 AVB: electrocardiographic atrioventricular block.

7 iRSB/RSB: (incomplete) right bundle block.

In the German Caucasians, the S377G allele frequency was not significantly different between ASDs, PFOs and TEE controls (range 12% to 13.5%), and it was close to the allele frequency of other world Caucasian populations (Table 5).

**Table 5:** GATA4 genotypes of German cohort.

		ASDII(n = 96)	PFO/Stroke(n = 95)	TEE Controls (no PFO/ASD)(n = 96)
**GATA4 S377G**	**Heterozygous (%)**	15 (15.6)	21 (22.1)	16 (16.7)
	**Homozygous (%)**	4 (4.2)	1 (1.1)	5 (5.2)
	**Allele Frequency**	12	12.1	13.5

### Pooled data

We continued recruitment of Australian Caucasians in the same St. Vincent's Hospital treatment population using identical inclusion criteria and patient classification as in the original study, but over two years were only able to recruit 32 new cases of PFO with neurologic event. Among them, only 8 had undergone PFO closure. This low number may be because of depletion of adults with severe PFO in the treatment population. However, in the same time frame, we collected an additional independent group of 134 TEE controls.

The power of both Australian and German studies was weakened by the low number of subjects. Given relative homogeneity of two studies, we pooled data from all subjects including the additional TEE controls, into one data set for analysis (Table 6). Subjects with PFO (n = 183) and ASD (n = 234) showed S377G allele frequencies of 13.9% and 10.7%, respectively, which were not significantly different compared to TEE controls (n = 340, S377G allele frequency = 11.9%).

**Table 6:** Pooled genotype data from Australian and German Cohorts.

		ASDII(n = 234)	PFO/Stroke(n = 183)	TEE Controls (no PFO/ASD)(n = 340)
**GATA4 S377G**	**Heterozygous (%)**	36 (15.4)	41 (22.4)	65 (19.1)
	**Homozygous (%)**	7 (3)	5 (2.7)	8 (2.4)
	**Allele Frequency**	10.7	13.9	11.9

## Discussion

GATA transcription factors regulate several biological processes during embryogenesis including cardiac development. Of the six vertebrate GATA factors, GATA4, GATA5 and GATA6 are involved in cardiogenesis [59]–[62]. Several mutations in GATA4 have been previously described in patients with CHD, including 677delC and Arg283His which were reported in patients with complex heart malformations encompassing ASD, VSD, common atrioventricular canal, and PFO [63]. S377G is located within the conserved and essential C-terminal GATA4 domain and leads to an increase in transcriptional activity of GATA4 *in vitro* [49]. Given the causal role of GATA4 in CHD, this common allele may play a modifier role.

We initially screened for GATA4 S377G in three different sets of CHD patients including those with ASD, PFO and other forms of CHD, all of Australian Caucasian origin. We observed high allele frequency of S377G in subjects with PFO and stroke (18%) which was significantly higher than in TEE controls in whom ASD and PFO were excluded (p = 0.02). This finding suggested a role for S377G in the etiology of PFO complicated by stroke. However, several observations make this result less compelling. Around one-fourth of Caucasians unselected for septal status would be expected to have PFO [14]. If S377G was involved in causation of PFO we would expect its frequency in Population Controls to be higher than that in TEE Controls. Indeed, this was the case (12.4% vs 9.3%). However the difference was not statistically significant (p = 0.20). Furthermore, the difference in S377G frequency between patients with PFO and stroke, and population controls (18.1% vs 12.4%) was non-significant.

It is possible that an increased frequency of S377G in the patients with PFO and stroke is due to an independent effect of this variant on stroke risk. For example, GATA4 represses the transcriptional activity of apolipoprotein(a) gene [64], high levels of which are an independent risk factor for premature atherosclerosis and stroke [65], [66]. However, a direct association between S377G and stroke is unlikely because the allele frequency of S377G in the “stroke/no PFO/ASD” group (12.1%) was not different from that of TEE or population controls.

There was no association between S377G and PFO complicated by cryptogenic stroke when we analysed an independent group of German Caucasians including 95 subjects with PFO, most of whom had experienced a neurologic event. The S377G allele frequency was not significantly different in any comparison between ASDs, PFOs and TEE controls. Analysis of combined Australian and German data confirmed the lack of association.

In our initial screen of 162 subjects (including 43 PFO/stroke, 9 incidental PFO with no stroke and 110 ASD subjects), all coding exons of the GATA4 gene were sequenced. No GATA4 variants were identified in the PFO/stroke cohort. However, we found 4 non-synonymous variants, 3 in the ASD group (full gene deletion; E359del; A411V) and one in an incidental PFO subject (D425N). These changes were not detected by subsequent screening in 270 controls. A411V and D425N have been reported in a separate publication describing a larger screen [67]. While potentially deleterious, there were no obvious functional consequences of these mutations in transient transfection assays.

In summary, the study of independent and pooled populations of Australian and German Caucasians failed to show a significant association between S377G and PFO with stroke. We conclude that the common GATA4 variant S377G is relatively benign in terms of its participation in CHD and PFO/Stroke. However, it remains possible that common mutations in other cardiac transcription factors play a significant role in the pathogenesis of cryptogenic stroke through causation of PFO.
